# Supplementary material for: The interactome of intact mitochondria by cross-linking mass spectrometry provides evidence for coexisting respiratory supercomplexes
Source: Mol Cell Proteomics. 2017 Dec 8;17(2):216–32. doi: 10.1074/mcp.RA117.000470 (PMC5795388; doi:10.1074/mcp.RA117.000470)
Supplement: Supplemental Data [file supp_RA117.000470_133922_0_supp_23978_fzffwf.zip › spectra_annotation/mito_spectra_annotation/221-1-8-1-7-1.pdf]

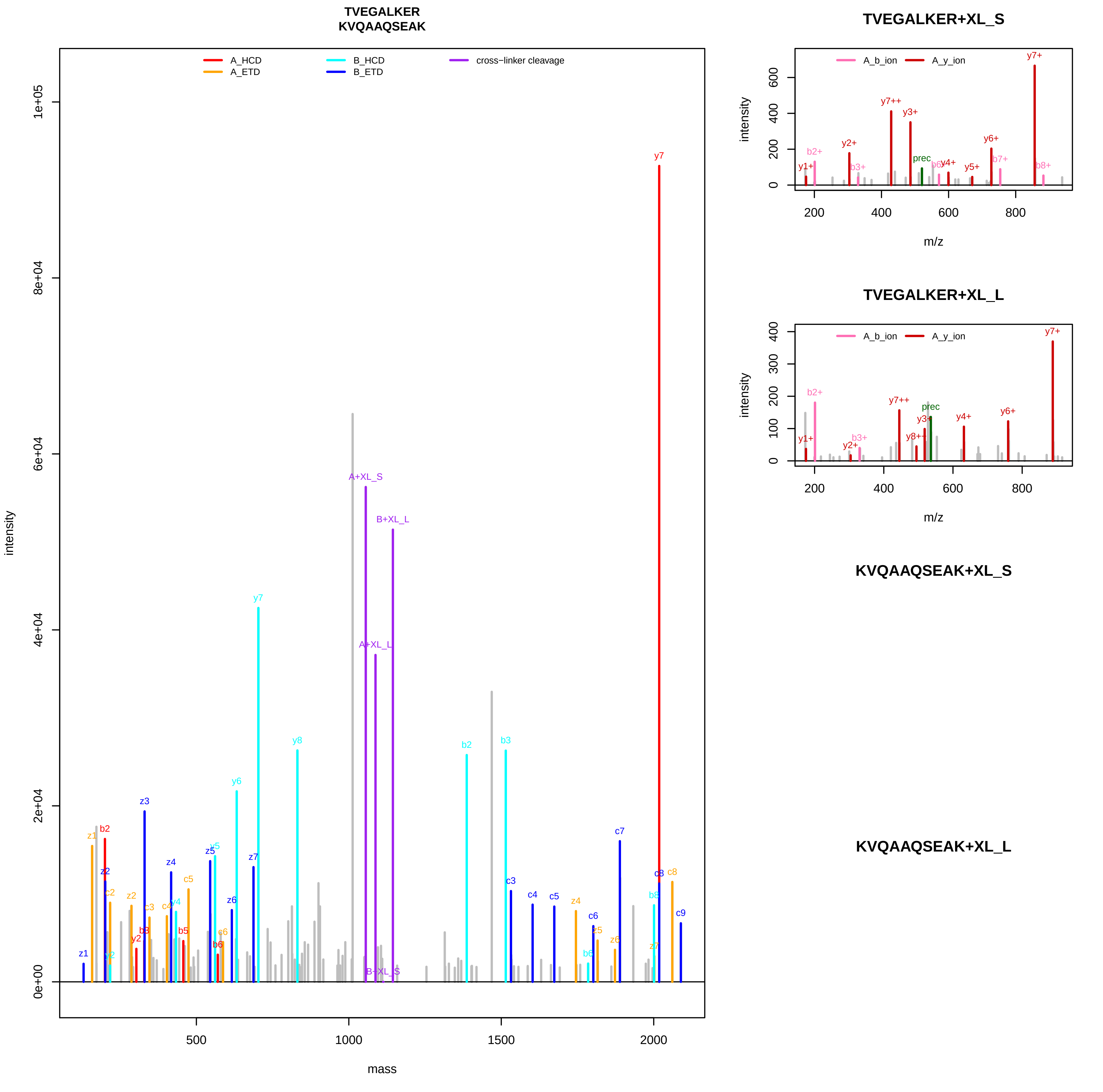

TVEGALKER
KVQAAQSEAK
A_HCD
A_ETD
B_HCD
B_ETD
cross−linker cleavage
intensity
mass
0e+00
2e+04
4e+04
6e+04
8e+04
1e+05
500
1000
1500
2000
y7
A+XL_S
B+XL_L
A+XL_L
B+XL_S
y8
b2
b3
c3
c4
c5
c7
c8
c9
z1
z2
z3
z4
z5
z6
z7
b2
b3
b5
b6
y2
c2
c3
c4
c5
c6
y5
y6
z4
z5
z6
z7
TVEGALKER+XL_S
A_b_ion
A_y_ion
intensity
m/z
y7+
y7++
y3+
y2+
b2+
y1+
b3+
prec
b6+
y4+
y5+
y6+
b7+
b8+
TVEGALKER+XL_L
A_b_ion
A_y_ion
intensity
m/z
y7+
b2+
y7++
y3+
y8++
prec
y4+
y6+
b3+
y2+
y1+
KVQAAQSEAK+XL_S
KVQAAQSEAK+XL_L
